# miRNA-seq of *Echinococcus multilocularis* Extracellular Vesicles and Immunomodulatory Effects of miR-4989

**DOI:** 10.3389/fmicb.2019.02707

**Published:** 2019-11-29

**Authors:** Juntao Ding, Guitian He, Jin’en Wu, Jing Yang, Xiaola Guo, Xing Yang, Ying Wang, Omnia M. Kandil, Ivan Kutyrev, Mazhar Ayaz, Yadong Zheng

**Affiliations:** ^1^College of Life Science and Technology, Xinjiang University, Urumqi, China; ^2^State Key Laboratory of Veterinary Etiological Biology, Key Laboratory of Veterinary Parasitology of Gansu Province, Lanzhou Veterinary Research Institute, Chinese Academy of Agricultural Sciences, Lanzhou, China; ^3^Department of Medical Microbiology and Immunology, School of Basic Medicine, Dali University, Dali, China; ^4^National Institute of Parasitic Diseases, Chinese Center for Disease Control and Prevention, Key Laboratory of Parasite and Vector Biology, Ministry of Health, National Center for International Research on Tropical Diseases, WHO Collaborating Center for Tropical Diseases, Shanghai, China; ^5^Departerment of Parasitology and Animal Disease, Veterinary Research Division, National Research Centre, Giza, Egypt; ^6^Institute of General and Experimental Biology, Siberian Branch of Russian Academy of Sciences, Ulan-Ude, Russia; ^7^Cholistan University of Veterinary and Animal Sciences, Bahawalpur, Pakistan; ^8^Jiangsu Co-innovation Center for Prevention and Control of Important Animal Infectious Diseases and Zoonoses, Yangzhou, China

**Keywords:** *Echinococcus multilocularis*, emu-miR-4989-3p, extracellular vesicles, macrophage, miRNA

## Abstract

Alveolar echinococcosis caused by *Echinococcus multilocularis* is an important zoonotic disease. In the infected mice, emu-miR-4989-3p is present in sera, but its role remains unknown. Using high-throughput sequencing and qPCR, emu-miR-4989-3p was herein confirmed to be encapsulated into *E. multilocularis* extracellular vesicles. In the transfected macrophages, emu-miR-4989-3p was demonstrated to significantly inhibit NO production compared to the control (*p* < 0.05). Moreover, transfection of emu-miR-4989-3p also gave rise to the increased expression of TNF-α (*p* < 0.01). Furthermore, emu-miR-4989-3p induced the dysregulation of several key components in the LPS/TLR4 signaling pathway compared with the control, especially TLR4 and NF-κB that both were upregulated. Conversely, the NO production and the expression of TNF-α, TLR4 and NF-κB tended to be increased and decreased in the mimics-transfected cells upon emu-miR-4989-3p low expression, respectively. These results suggest that emu-miR-4989-3p is one of ‘virulence’ factors encapsulated into the extracellular vesicles, potentially playing a role in the pathogenesis of *E. multilocularis*.

## Introduction

*Echinococcus multilocularis* is a small tapeworm responsible for alveolar echinococcosis in human beings. Adult worms reside in the intestine of foxes, dogs and wolves, and eggs are expelled with the feces into surrounding environment. Eggs develop into protoscolex-containing cysts mainly in the liver of rodents who consume egg-contaminated food or water. When foxes prey on the infected rodents, protoscoleces develop into the adults in the intestine, thus finishing its life cycle. Occasionally, humans are infected with eggs and experience a long latent period without clinical symptoms. Due to the tumor-like growth of the parasite, the disease is lethal if not properly treated or untreated. It was estimated that the disability adjusted life years caused by this disease was up to 687,823 in 2010 (Torgerson et al., 2015).

During infection, parasites use various strategies to change the host environment to improve their survival and escape the host immune system, and the dynamic release of extracellular vesicles is one of the strategies (Ofir-Birin and Regev-Rudzki, 2019). Extracellular vesicles are lipid bilayer-enclosed entities with a size from 30 to 1000 nm in diameter, and they contain a variety of bioactive molecules, including lipids, glycans, proteins, microRNAs and DNA. Of them, microRNAs (miRNAs) are a subclass of small regulatory RNAs, which bind to specific sites in the untranslated region and then induce translational repression or degradation of target mRNA, and they are closely associated with the pathogenesis of parasites (Zheng et al., 2013). MiR-4989 belongs to the miR-277 family, and it has only been found in protostomes up to date (Fromm et al., 2013). In parasitic flatworms, miR-4989 is organized together with miR-277 to form a miRNA cluster (Jin et al., 2013; Cucher et al., 2015; Protasio et al., 2017). High-throughput sequencing results revealed that miR-4989 was one of the highest expressed miRNAs in all cestodes investigated (Cucher et al., 2015; Basika et al., 2016; Zheng, 2017). Most recently, miR-4989 was shown to be upregulated in adult *Schistosoma mansoni* and localized to the entire tegument of male worms, suggesting a role in the development (Protasio et al., 2017). This finding also favors an idea that sma-miR-4989 may be secreted by extracellular vesicles just like sma-miR-277 (Nowacki et al., 2015; Protasio et al., 2017). In agreement with this hypothesis, our recent study showed that both *E. multilocularis* miR-4989-3p and -277 were present in the sera of infected mice (Guo and Zheng, 2017), but their role remains elusive. It is possible that these circulating emu-miR-4989-3p and -277 are also released by extracellular vesicles that are potentially involved in parasitic infections (Buck et al., 2014; Cwiklinski et al., 2015; Szempruch et al., 2016; Zhu et al., 2016; Guo and Zheng, 2017).

High-throughput sequencing and qPCR results herein verified that emu-miR-4989-3p was encapsulated into the extracellular vesicles produced by *E. multilocularis*. It was also shown a role of emu-miR-4989 in regulation of nitric oxide (NO) production, and expression of TNF-α and several key components involving in the LPS/TLR4 signaling pathway in macrophages, suggesting an immunoregulatory role in *E. multilocularis* infection.

## Materials and Methods

### Cells and Transfection

RAW264.7 macrophages were cultured in RPMI-1640 with addition of 10% fetal bovine serum (Invitrogen) at 37°C, 5% CO_2_. Cells with 70–80% confluence were harvested and their viability was checked by trypan-blue exclusion (Sigma). Approximately 0.5 × 10^6^ cells were seeded into each well in 6-well plates (Costar, Washington, United States) and cultured until the confluence reached 70–80%. Then cells were subjected to transfection with emu-miR-4989-3p mimics, an artificially synthesized RNA that mimics endogenous precursor emu-miR-4989-3p, at a final concentration of 30 nM using RNAiMAX (Invitrogen) according to the instructions. Briefly, RNAiMAX Reagent and mimics or inhibitor were diluted in Opti-MEM Medium (Gibco), respectively, then mixed thoroughly (1:1 ratio), and incubated for 5 min at room temperature. Then the mixture was added into cells and incubated at 37°C, 5% CO_2_. Twelve h later after transfection, to further verify the functions of exogenetic emu-miR-4989-3p, the mimics-transfected cells were then transfected with emu-miR-4989-3p inhibitor and cultured for another 12 h. Subsequently, the media was replaced by fresh RPMI-1640 with 100 ng/mL LPS (Sigma) and 10 ng/mL IFN-γ (Sigma). Twenty-four h later, culture supernatant and cells were collected for downstream analysis. Prior to RNA extraction, cells were briefly washed in ice-cold DEPC-treated PBS three times. In these experiments, negative control constructs (Invitrogen) were used as a control.

### Total RNA Extraction

Total RNA for small RNA sequencing library construction and qPCR analysis was extracted from macrophages, extracellular vesicles and *E. multilocularis* protoscoleces, which the last two were previously prepared and stored at −80°C in our lab (Zheng et al., 2017), using TRIzol LS Reagent (Invitrogen) according to the instructions with some modifications. Briefly, RNA-containing aqueous supernatant was added with 1 μL glycogen (10 mg/mL, Invitrogen) and then subjected to incubation overnight at −20°C after homogenization and centrifugation. Pellets were air-dried and resuspended into nuclease-free water. RNA concentration and quality were evaluated using Bioanalyzer (Agilent). Recovered RNA samples were immediately used or stored at −80°C for late use.

### High-Throughput Sequencing and Data Analysis

To determine whether emu-miR-4989-3p is released via extracellular vesicles, their small RNA cargo was determined by deep sequencing. *E. multilocularis* extracellular vesicles were previously prepared and stored at −80°C in our lab (Zheng et al., 2017). Prior to RNA extraction, extracellular vesicles were resuspended and treated with RNase A (Invitrogen) at 37°C for 30 min for removal of RNA contaminants. A small RNA sequencing library was constructed and sequenced as previously described (Jin et al., 2017). The process of raw sequencing data was performed according to the previously reported methods with some modification (Zheng, 2017). In brief, after removal of the reads with low quality and less than 18 nt, the ‘clean’ reads were mapped to the full annotated genome of *E. multilocularis*^1^. Afterward, all the mapped reads were used for identification of miRNAs (miRBase^2^), tRNAs, rRNAs, snoRNAs and snRNAs (snoRNABase^3^), repetitive sequences and small degraded mRNA (GenBank^4^) by blast searching against individual databases. For identification of potential novel miRNAs, miRDeep was used with default parameters.

### NO Determination

The supernatant of the macrophage culture was collected at 12 and 24 h after transfection, respectively. The levels of NO in the supernatant were detected using Griess reagent (Invitrogen) according to the instructions with some modifications. First, samples were thoroughly mixed with fresh-prepared Griess reagent and nuclease-free water in a 96-well plate (Eppendorf). After light-proof incubation, the absorbance values at 570 nm were read by a microplate reader (Bio-Rad). Each of samples was assessed in triplicate and the NO levels were calculated using the results from three independent experiments.

### qPCR Analysis

For miRNA qPCR, complementary DNA (cDNA) was synthesized from 1 μg (with the addition of approximately 267 fmol of *Caenorhabditis elegans* miR-39 (QIAGEN) as an exogenous reference) using All-in-One miRNA First Strand cDNA Synthesis Kit (GeneCopoeia) with a RT primer (Table 1).

**TABLE 1:** The primers used in this study.

**Primer**	**Sequence (5′-3′)**
RT primer	GCGAGCACAGAATTAATACGAC TCACTATAGG(T)_12_VN^*a*^
universal reverse primer	GCGAGCACAGAATTAATACGAC
tRNA-derived sRNA	GCCTTCGTAGCTCAGGGG
emu-miR-4898-3p	AAAATGCACCAACTATCTGAG
emu-miR-190-5p	GAGATATGTTTGGGTTACTTG
emu-miR-219-5p	TGATTGTCCATTCGCATTTC
emu-miR-61-3p	TGACTAGAAAGAGCACTCAC
emu-miR-71-5p	GCTGAAAGACGATGGTAGTG
emu-let-7-5p	GCTGAGGTAGTGTTTCGAATGTCT

*^*a*^‘V’ stands for A, G, or C; ‘N’ stands for A, T, G or C.*

For qPCR of iNOS, cytokines and key genes in the LPS/TLR4 signaling pathway, 1.5 μg of total RNA were used for cDNA synthesis using RevertAid First Strand cDNA Synthesis Kit (Thermo Fisher Scientific) with Oligo d(T)_18_ according to the instructions. After reaction, cDNA products were diluted by addition of 7 volumes of nuclease-free water, and immediately used for qPCR or stored at −20°C.

To verify the high-throughput RNA sequencing, tRNA-derived sRNA and six miRNAs including emu-miR-71-5p, -4898-3p, -190-5p, -219-5p, -61-3p and -let-7-5p were selected for qPCR analysis with specific primers (Table 1). To evaluate the effects of emu-miR-4989-3p on the LPS/TLR-4 signaling pathway, eleven key genes including *cd14*, *tlr4*, *myd88*, *tirap*, *ticam1*, *ticam2*, *irf-3*, *irf-5*, *ripk1*, *nf-*κ*B* and *ap-1* were selected. For cytokines, two anti-inflammatory cytokine genes *il-4* and *il-10* as well as five pro-inflammatory cytokine genes *tnf-*α, *il-1*α, *il-1*β, *il-6* and *il-12B* were selected. All the primers including the primers for *inos* were validated and purchased from GeneCopoeia. The qPCR was conducted as previously reported (Guo and Zheng, 2017) and reaction was conducted on ABI 7500 real-time PCR system (ThermoFisher Scientific). The relative gene expressions of miRNA and mRNA were calculated using the 2^–ΔΔ*Ct*^ formula method and normalized to that of cel-miR-39 and GAPDH, respectively. Each of genes was assessed in triplicate and the final expression levels were calculated using the results from three independent experiments.

### Western Blotting

Total proteins were extracted from treated macrophages cells using RIPA lysis buffer containing proteinase inhibitor (Sigma). The protein concentration was determined by Bradford. An equivalent amount of proteins (30 μg/lane) was separated onto 12% sodium dodecyl sulfate-polyacrylamide gel electrophoresis (SDS-PAGE), and transferred to PVDF membrane. The membranes were blocked using 5% bovine serum albumin (Sigma) in PBS containing 0.1% Tween-20 at room temperature for 1h, and were then incubated at 4°C overnight with the primary antibodies TLR4 (1:400, Invitrogen), NF-κB p105/p50 (1:1000, Abcam) and rabbit anti-GAPDH (1:1000, Sigma), respectively. After several washes in PBST, the membranes were incubated with horseradish peroxidase (HRP)-conjugated anti-rabbit IgG (H + L) (1:10000, SeraCare). The protein signals were visualized by exposure to X-ray films using an enhanced chemiluminescence system (ECL) and analyzed by ImageJ.

### ELISA

The levels of TNF-α in culture supernatant were assessed using DuoSet ELISA Kit (R&D Systems) strictly according to the manufacturer’s instructions. Absorbance values were read at 450 nm using a microplate reader (Bio-Rad). Each sample was tested in triplicate.

### Statistical Analysis

Using GraphPad Prism 5 software, statistical analysis was performed with a two-tailed unpaired *t*-test for comparison of the difference between two groups and with one-way ANOVA for comparison of the difference among four groups. A *p* value less than 0.05 was considered to be significantly different.

## Results

### Small RNA Profile of *E. multilocularis* Extracellular Vesicles

**FIGURE 1:**
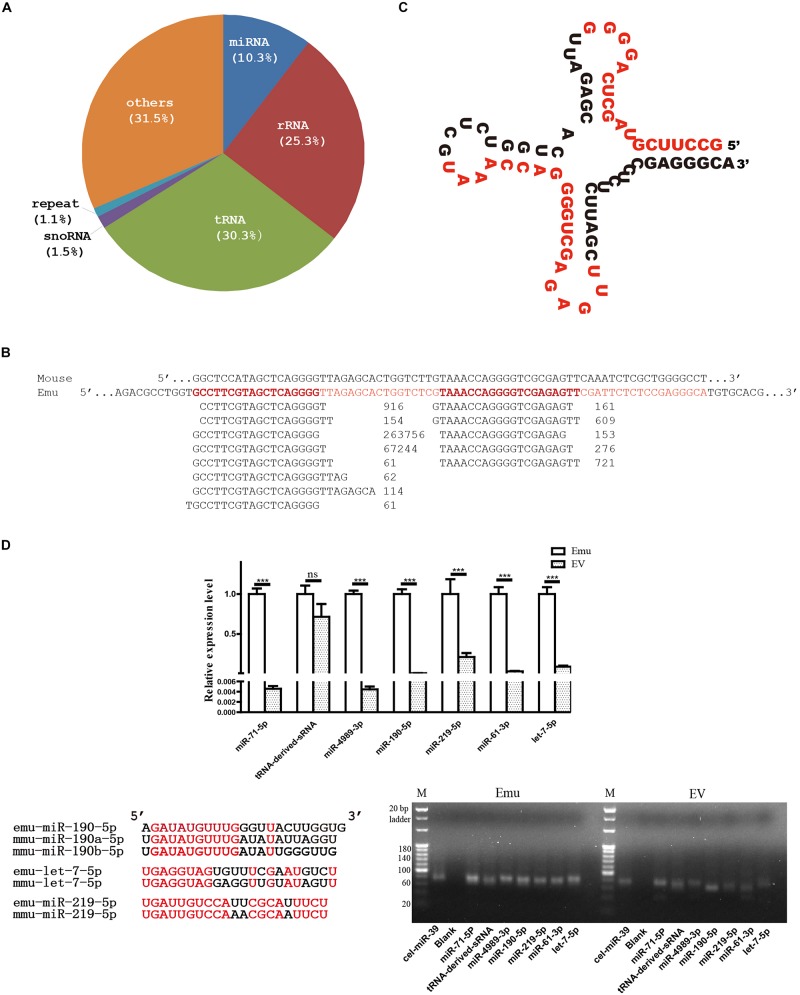
Small RNA profile of *E. multilocularis* extracellular vesicles. **(A)** Catalogs of small RNA identified. **(B)** Alignment of threonine tRNA-derived small RNAs. Short reads were aligned beneath the threonine tRNA gene of *E. multilocularis* (Emu, in red), and read counts were placed after each sequence. For comparison, mouse threonine tRNA gene (Mouse) was aligned together. **(C)** Secondary structure of threonine tRNA gene of *E. multilocularis*. Two short sequences with the highest read count in each of two mapped read sets were highlighted in red. **(D)** qPCR validation and electrophoresis of tRNA-derived small RNA (sRNA) and six miRNAs. Using *C. elegans* miR-39 as a exogenous reference, their abundance was comparatively evaluated in both *E. multilocularis* metacestodes (Emu) and extracellular vesicles (EV). Of six *E. multilocularis* miRNAs, only three, including emu-miR-190-5p, emu-let-7-5p and emu-219-5p, have orthologs in mice and the identical nucleotides are shown in red in the alignment. ^∗∗∗^*p* < 0.001, NS: not significant.

In total, 24,599,107 reads were obtained, which were cataloged into 183,709 unique reads. There were 1,264,544 reads mapped to the genome of *E. multilocularis*, which were grouped into 6,509 unique reads, and approximately 68.5% of the mapped reads were annotated. Of the annotated reads, nearly 30.3% were identified as tRNA-derived small RNAs (sRNA), whereas 25.3% were found to be of rRNA origin and 10.3% were identified as miRNAs (Figure 1A).

Approximately 87.2% of 383,527 tRNA-derived reads were originated from threonine tRNA of *E. multilocularis* (Figure 1B), and the positions of two mapped sequence sets were relatively fixed (Figure 1C). Obviously, the majority of the threonine tRNA-derived small sequences were positioned on the 5′ end, and one sequence was predominant with 263,756 read counts (Figure 1B).

### emu-miR-4989 Encapsulated Into *E. multilocularis* Extracellular Vesicles

A total of 130,612 mapped reads were annotated as miRNAs, leading to identification of 18 different miRNAs that belonged to 16 distinct miRNA families (Table 2). Distinct miRNAs were dramatically different in abundance, from emu-miR-71-5p with 47,742 read counts to emu-miR-307-3p with 45. Moreover, four miRNAs including emu-miR-71-5p, -let-7-5p, -miR-4989-5p and -miR-10-5p were dominantly represented, accounting for 78.5% of total miRNA reads (Table 2).

**TABLE 2:** miRNAs encapsulated into *E. multilocularis* extracellular vesicles.

**miRNA**	**Read count**	**Sequence (5′-3′)**	**Length**	**Family**
emu-miR-71-5p	47,742	UGAAAGACGAUGGUAGUGAGA	21	miR-71
emu-let-7-5p	22,860	UGAGGUAGUGUUUCGAAUGUCU	22	let-7
emu-miR-4989-5p	16,157	UGGGUAGUCGUUGCAUUUCC	20	miR-277
emu-miR-10-5p	15,770	CACCCUGUAGACCCGAGUUUG	21	miR-10
emu-miR-4989-3p	8,067	AAAAUGCACCAACUAUCUGAGA	22	miR-277
emu-miR-124b-3p	5,246	UUAAGGCACGCGGUGAAUGCCAA	23	miR-124
emu-miR-7a-5p	2,940	UGGAAGACUGGUGAUAUGUUGU	22	miR-7
emu-miR-125-5p	2,767	UCCCUGAGACCCUAGAGUUGUC	22	miR-125
emu-miR-219-5p	2,420	UGAUUGUCCAUUCGCAUUUCU	21	miR-219
emu-miR-61-3p	2,035	UGACUAGAAAGAGCACUCACA	21	miR-279
emu-miR-1-3p	1,627	UGGAAUGUUGUGAAGUAUGU	20	miR-1
emu-miR-190-5p	948	AGAUAUGUUUGGGUUACUUGGUG	23	miR-190
emu-miR-184-3p	913	GGGACGGAAGUCUGAAAGGUUU	22	miR-184
emu-miR-2a-3p	528	AAUCACAGCCCUGCUUGGAAC	21	miR-2
emu-miR-87-3p	269	GUGAGCAAAGUUUCAGGUGU	20	miR-87
emu-miR-2c-3p	193	CACAGCCAAUAUUGAUGAAC	20	miR-2
emu-miR-31-5p	85	UGGCAAGAUACUGGCGAAGCU	21	miR-31
emu-miR-307-3p	45	UCACAACCUACUUGAUUGAGGGG	23	miR-67

In line with the sequencing data, the qPCR and electrophoresis results confirmed that tRNA-derived-sRNA and six miRNAs were present in extracellular vesicles. However, except tRNA-derived-sRNA, all the miRNAs tested were expressed at a significantly lower level in extracellular vesicles than that in *E. multilocularis* metacestodes (Figure 1D).

**FIGURE 2:**
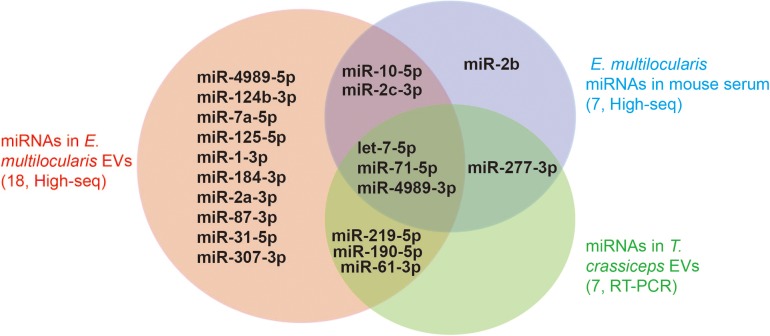
miRNAs identified in *E. multilocularis* extracellular vesicles. Extracellular vesicle miRNA cargoes encompassed the majority of miRNAs that were identified in the sera of *E. multilocularis*-infected mice by high-throughput sequencing (High-seq), and in *T. crassciceps* extracellular vesicles by reverse-transcription PCR (RT-PCR). The numbers in brackets stand for the total number of miRNAs identified in each study. EVs, extracellular vesicles.

Canonical mature emu-miR-4989, emu-miR-4989-3p, were also found in the extracellular vesicles, but its abundance was remarkably lower compared with that in the larvae (*p* < 0.001, Figure 1D). Consistently, comparative results revealed that, except miR-2b and miR-277-3p, all the remaining parasite miRNAs circulating in *E. multilocularis*-infected mouse sera or loaded into *Taenia crassiceps* extracellular vesicles were present in *E. multilocularis* extracellular vesicles (Figure 2). Moreover, three miRNAs emu-miR-4989-3p, -let-7-5p and -miR71-5p were commonly shared (Figure 2).

### Immunomodulatory Effects of emu-miR-4989-3p

**FIGURE 3:**
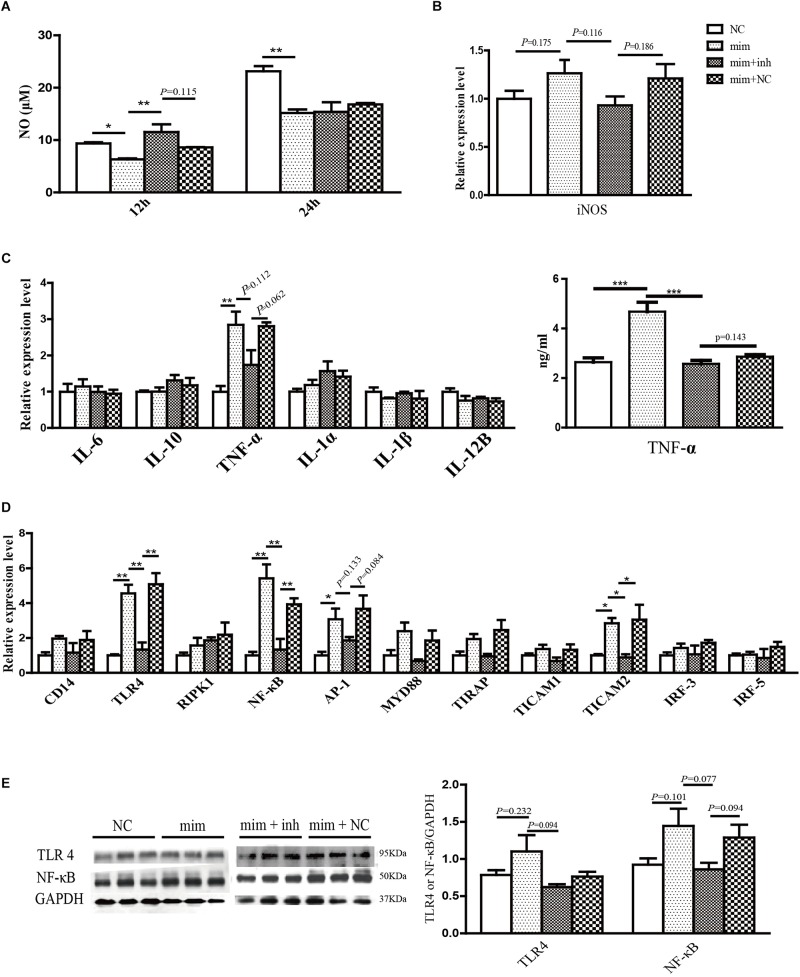
Immunoregulatory effects of emu-miR-4989-3p in the transfected RAW264.7 macrophages. **(A)** Analysis of NO secretion in the control (NC group), the emu-miR-4989-3p mimics-transfected cells (mim group), the emu-miR-4989-3p mimics-transfected cells after emu-miR-4989-3p inhibitor transfection (mim + inh group) and the emu-miR-4989-3p mimics-transfected cells after control construct transfection (mim + NC group). Data are expressed as mean ± s.e.m.; ^∗^*p* < 0.05, ^∗∗^*p* < 0.01. Data for the final analysis are from three independent experiments. **(B)** qPCR analysis of *inos* gene expression. Data are expressed as mean ± s.e.m. Data for the final analysis are from three independent experiments. **(C)** Analysis of the expression of cytokine genes by qPCR (left panel) and TNF-α by ELISA (right panel). Data are expressed as mean ± s.e.m. ^∗∗^*p* < 0.01, ^∗∗∗^*p* < 0.001. Data for the final analysis are from three independent experiments. **(D)** Analysis of the expression of eleven key genes on the LPS/TLR4 signaling pathway by qPCR. Data are expressed as mean ± s.e.m.; ^∗^*p* < 0.05, ^∗∗^*p* < 0.01. Data for the final analysis are from three independent experiments. **(E)** Western blotting analysis of TLR4 and NF-κB. Data are expressed as mean ± s.e.m.

In the activated macrophages, emu-miR-4989-3p was shown to significantly suppress NO secretion at 12 h and 24 h after transfection compared with the control (*p* < 0.05, Figure 3A). However, the expression of *inos*, a gene involved in NO production, was not significantly elevated in the cells transfected with emu-miR-4989-3p mimics (*p* = 0.175, Figure 3B).

After transfection of emu-miR-4989-3p mimics, except *tnf-*α, all the cytokine genes had no significant change in expression. The TNF-α was remarkably upregulated at the mRNA and protein levels in the emu-miR-4989-3p-treated cells compared with that in the control-treated cells (*p* < 0.01, Figure 3C). In both control- and emu-miR-4989-3p-treated macrophages, the levels of *il-4* mRNA were too low to be detected (data not shown).

Similarly, emu-miR-4989-3p was also shown to be able to disturb the expression of key genes involved in the LPS/TLR-4 signaling pathway in the treated macrophages. Compared to the control, emu-miR-4989-3p was found to induce overexpression of *tlr4*, *nf-*κ*B*, *ap-1* and *ticam2* (*p* < 0.05, Figure 3D). Western blot analysis also confirmed that emu-miR-4989-3p induced a slight increase of TLR4 (*p* = 0.232) and NF-κB (*p* = 0.101) at a protein level compared with the control (Figure 3E).

As expected, the NO production and the expression of TNF-α, TLR4 and NF-κB tended to be increased and decreased in the mimics-transfected cells after inhibitor transfection (mim + inh groups, Figures 3A–E), respectively.

## Discussion

Using high-throughput sequencing, a total of 18 different miRNAs were confirmed as a part of RNA cargo in the extracellular vesicles in this study, being less than 37 mature miRNAs identified in *E. multilocularis* metacestodes (Cucher et al., 2015). In agreement with the previous findings, this miRNA data set encompassed the vast majority of parasite miRNAs that were detected in the sera of *E. multilocularis*-infected mice (Guo and Zheng, 2017) and in *T. crassciceps* extracellular vesicles (Ancarola et al., 2017). Of 18 different miRNAs, some including emu-let-7-5p, -miR-10-5p, -miR-71-5p and -miR-4989-3p were abundant in the extracellular vesicles as well as in parasites. However, some miRNAs that were relatively highly expressed in *E. multilocularis* metacestodes were absent in the extracellular vesicles, such as emu-bantam-3p and -miR-9-5p (Cucher et al., 2015). These results suggest that parasites selectively encapsulate miRNA cargo into extracellular vesicles.

In addition to miRNAs, one group of threonine tRNA-derived small RNAs was highly abundant in the extracellular vesicles, one of which the read count accounted for 68.7% of total counts of tRNA-derived small sequences. Moreover, their mapped positions were rather fixed, being mainly at the 5′ most end of threonine tRNA, suggesting that these sequences do not result from degradation. Consistent with this idea, the qPCR and electrophoresis results verified its expression in both parasite and extracellular vesicles. At present, such tRNA-derived small RNAs have also been reported in the extracellular vesicles secreted by other parasites (Lambertz et al., 2015; Nowacki et al., 2015). In mice, tRNA-derived small RNAs were recently shown to be involved in ribosome biogenesis by promoting ribosomal protein mRNA translation (Kim et al., 2017), and in genome stability by the repression of retrotransposon mobility (Schorn et al., 2017). In *Giardia lamblia*, such tRNA-derived small RNAs were supposed to be related to the differentiation (Liao et al., 2014). Due to the lack of Piwi subfamily AGOs (Zheng, 2013), *E. multilocularis* is supposed not to express Piwi-interacting RNAs (piRNAs), and these tRNA-derived small RNAs may replace piRNAs to protect the genome from transposon integration. The biological functions of tRNA-derived small RNAs in the extracellular vesicles remain to be established. Of high interest is to decipher their role in the parasite-host interactions in future.

In this study, emu-miR-4989 was confirmed to be encapsulated into *E. multilocularis* extracellular vesicles. Moreover, canonical mature emu-miR-4989-3p together with its counterpart, emu-miR-4989-5p, co-existed in the extracellular vesicles, but the former had less abundance than the latter. In *E. multilocularis*, their abundance is just reversed (Cucher et al., 2015). Normally, the counterparts of mature miRNAs are subjected to degradation, but some really enter into small RNA-induced silencing networks in a few cases (Zheng et al., 2013). It is possible that emu-miR-4989-5p in the extracellular vesicles is functional, and it is required to investigate its role in future studies.

As parasite extracellular vesicles can be internalized by host cells or organs (Buck et al., 2014; Szempruch et al., 2016; Zhu et al., 2016), encapsulated emu-miR-4989-3p is deduced to be associated with the pathogenesis of *E. multilocularis*. Indeed, it was shown that emu-miR-4989-3p remarkably suppressed NO production in the stimulated macrophages after transfection. It is well known that NO is one of crucial determinants that affect the consequences of *Echinococcus* species infections (Vuitton and Gottstein, 2010). It has already been found that several molecules are likely to be involved in regulation of NO production (Andrade et al., 2004; Zheng et al., 2016). Our recent study demonstrated that *E. multilocularis* extracellular vesicles were capable of repressing NO secretion (Zheng et al., 2017). It is feasible that encapsulated emu-miR-4989-3p is, at least partially, responsible for this repression. Furthermore, emu-miR-4989-3p was demonstrated to be capable of modulating the expression of cytokine genes and the genes essential for LPS/TLR4 signaling transduction. In the transfected macrophages, although anti-inflammatory cytokine gene *il-10* seemed to be stably expressed, pro-inflammatory cytokine TNF-α was remarkably upregulated, suggesting emu-miR-4989-3p acting as an activator of inflammation. Similarly, the expression of several key genes in the LPS/TLR4 signaling pathway was dramatically elevated. These immunomodulatory effects are similar to the observations in the same cells treated with *E. multilocularis* extracellular vesicles (Guo and Zheng, 2017), indicating that encapsulated emu-miR-4989-3p is one of effector molecules in the extracellular vesicles. To better understand its role in the parasite-host interplay, it is necessary to *in vivo* evaluate the biological activities and the immunomodulatory effects of encapsulated emu-miR-4989-3p in future studies.

## Conclusion

Using high-throughput sequencing and qPCR, emu-miR-4989-3p was validated to be loaded into *E. multilocularis* extracellular vesicles. In the transfected RAW264.7 macrophages, emu-miR-4989-3p was demonstrated to be capable of modulating NO production, and the expression of cytokine genes and the key components in the LPS/TLR4 signaling pathway. These results suggest the modulatory capacity of emu-miR-4989-3p, which potentially plays a role in the pathogenesis of *E. multilocularis*.

## Data Availability Statement

The datasets generated in this study have been deposited into the BioProject database (accession: PRJNA579248).

## Author Contributions

YZ designed the experiments. JD, GH, JW, JY, XG, XY, and YW performed the experiments. JD, GH, OK, IK, and MA analyzed the data. YZ and GH wrote the manuscript.

## Conflict of Interest

The authors declare that the research was conducted in the absence of any commercial or financial relationships that could be construed as a potential conflict of interest.

## Footnotes

1 https://www.genedb.org/#/species/Emultilocularis

2 http://www.mirbase.org/index.shtml

3 https://www-snorna.biotoul.fr/index.php

4 ftp://ftp.ncbi.nlm.nih.gov/genbank/
